# Supplementary material for: Common data elements for predictors of pediatric sepsis: A framework to standardize data collection
Source: PLoS One. 2021 Jun 10;16(6):e0253051. doi: 10.1371/journal.pone.0253051 (PMC8192005; doi:10.1371/journal.pone.0253051)
Supplement: S1 Appendix — (PDF) [file pone.0253051.s002.pdf]

S1 Appendix. Search Strategy for Literature Review

1. exp Sepsis/
2. *sepsis.mp.*
3. 1 or 2
4. exp Triage/
5. prediction.mp.
6. warning score.mp.
7. risk factor*.mp.
8. or/4-7
9. 3 and 8
10. global health/
11. developing countr*.mp.
12. resource limited settings*.mp.
13. or/10-12
14. 9 and 13
15. exp child/ or exp infant/
16. 14 and 15
