# Supplementary material for: Common data elements for predictors of pediatric sepsis: A framework to standardize data collection
Source: PLoS One. 2021 Jun 10;16(6):e0253051. doi: 10.1371/journal.pone.0253051 (PMC8192005; doi:10.1371/journal.pone.0253051)
Supplement: S1 Table — (PDF) [file pone.0253051.s003.pdf]

**S3 Table. Preliminary list of predictor variables compiled through literature review (N=144). Displays decision to include (N=77) or exclude (N=67) from the common data element set, and reason for exclusion where applicable.**

| Variable | Step 1 | Step 2 | Reason for Exclusion | Tier |
|---|---|---|---|---|
| **Patient Characteristics** | | | | |
| Age | Included | | | 1 |
| Gender | Included | | | 1 |
| Time since last hospitalization | Included | | | 2 |
| Duration of illness/sign/symptom | Excluded | | Low perceived predictive value | |
| Referral (urgent) | Included | | | 2 |
| Distance from hospital | Discussion | Excluded | Low perceived predictive value | |
| Immunizations up to date | Discussion | Included | | 3 |
| History of no cry at birth | Excluded | | Low perceived predictive value | |
| History of feeding problem since birth | Included | | | 2 |
| **Pregnancy/Birth Details** | | | | |
| Born at home | Discussion | Excluded | Captured in "Facility of Birth" variable | |
| Facility of birth (reported) | Discussion | Included | | 2* |
| Attendant at birth (reported) | Included | | | 2* |
| Delivered by a skilled attendant | Discussion | Excluded | Low perceived predictive value | |
| Duration of labour | Excluded | | Low perceived predictive value | |
| Duration of rupture of membrane | Excluded | | Low perceived predictive value | |
| Premature rupture of membranes | Excluded | | Low perceived predictive value | |
| Ballard score | Excluded | | Measurement reliability | |
| Number of postnatal care visits (reported) | Included | | | 2* |
| **Sociodemographic Information** | | | | |

| | | | | |
|---|---|---|---|---|
| Mother alive | Included | | | 2 |
| Maternal age (years) | Included | | | 2 |
| Maternal education | Included | | | 2 |
| Maternal fever | Excluded | | Low perceived predictive value | |
| Maternal persistent cough | Excluded | | Low perceived predictive value | |
| Maternal HIV | Included | | | 1 |
| Child HIV status | Included | | | 1 |
| Exclusive breastfeeding | Included | | | 2 |
| Number of children in family | Excluded | | Low perceived predictive value | |
| Siblings death | Excluded | | Low perceived predictive value | |
| Bed-net use | Discussion | Included | | 2* |
| Consider malaria risk | Excluded | | Measurement reliability | |
| Boil all drinking water | Discussion | Included | | 3* |
| Water source for drinking (reported) | Discussion | Included | | 3* |
| Latrine available (reported) | Discussion | Included | | 3* |
| **Anthropometric Data** | | | | |
| Weight | Included | | | 1 |
| Length | Included | | | 1 |
| MUAC | Included | | | 1 |
| **Vitals** | | | | |
| Temperature | Included | | | 1 |
| HR | Included | | | 1 |
| RR | Included | | | 1 |
| SpO2 | Included | | | 1 |
| SBP (raw) | Discussion | Excluded | Low perceived predictive value | |
| DBP (raw) | Discussion | Excluded | Low perceived predictive value | |

| **Clinical Signs/Symptoms** | | | | |
|---|---|---|---|---|
| **Respiratory** | | | | |
| Apnea (reported) | Excluded | | Low perceived predictive value | |
| Apnea (observed) | Excluded | | Low perceived predictive value | |
| Difficulty Breathing (reported) | Included | | | 1 |
| Difficulty breathing (observed) | Included | | | 1 |
| Fast breathing (reported) | Excluded | | Measurement reliability | |
| Fast breathing (observed) | Excluded | | Measurement reliability | |
| Runny nose (reported) | Included | | | 3* |
| Respiratory effort (observed) | Excluded | | Measurement reliability | |
| Obstructed breathing (observed) | Included | | | 2 |
| Central cyanosis (observed) | Included | | | 2 |
| Chest in-drawing (observed) | Included | | | 2 |
| Nasal flaring (observed) | Included | | | 2 |
| Grunting (observed) | Included | | | 2 |
| Head bobbing/nodding (observed) | Included | | | 2 |
| Crepitations (observed) | Included | | | 2 |
| Stridor (observed) | Included | | | 2 |
| Wheezing (observed) | Included | | | 2 |
| Cough (reported) | Excluded | | Low perceived predictive value | |
| **Circulation/Perfusion** | | | | |
| Capillary Refill Time | Excluded | | Measurement reliability | |
| Prolonged capillary refill | Included | | | 1 |
| Skin cold (cool peripheries) | Included | | | 2 |
| Weak and fast pulse | Included | | | 2 |
| Pallor - palmar, oral, conjunctival | Included | | | 2 |

| | | | | |
|---|---|---|---|---|
| Mother feels baby is cold | Included | | | 3 |
| **Dehydration** | | | | |
| WHO dehydration scale | Excluded | | Measurement reliability | |
| Pinching of the skin of abdomen/hand (skin turgor) | Included | | | 2 |
| Sunken eyes | Included | | | 2 |
| Depressed fontanelle | Included | | | 2 |
| No tears when crying | Included | | | 2 |
| Reduced urine production | Excluded | | Low perceived predictive value | |
| Thirst | Discussion | Excluded | Measurement reliability | |
| Dry oral mucosa | Included | | | 2 |
| **Neurological** | | | | |
| Irritability (restlessness) (reported) | Excluded | | Measurement reliability | |
| Consolability (reported) | Excluded | | Measurement reliability | |
| Change in level of activity (reported) | Excluded | | Measurement reliability | |
| Change in crying (reported) | Excluded | | Measurement reliability | |
| Quality of crying (reported) | Excluded | | Measurement reliability | |
| Sleepiness/drowsiness/unconscious (reported) | Excluded | | Measurement reliability | |
| Ease of awakening (reported) | Excluded | | Measurement reliability | |
| Convulsions (reported) | Included | | | 2 |
| Convulsions (observed) | Included | | | 2 |
| Limbs became limp (reported) | Excluded | | Low perceived predictive value | |
| Neck pain/stiffness (reported) | Included | | | 2 |
| Stiff neck (observed) | Included | | | 2 |
| Stiff limbs (observed) | Included | | | 2 |
| Hypotonia (observed) | Included | | | 2 |
| Not able to drink or feed anything (reported) | Included | | | 2 |

| | | | | |
|---|---|---|---|---|
| Not feeding well (observed) | Excluded | | Low perceived predictive value | |
| Not suckling/breastfeeding (reported) | Included | | | 2 |
| Quality of suck (reported) | Excluded | | Measurement reliability | |
| Prostration (reported) | Excluded | | Measurement reliability | |
| Spontaneous movements/to stimulus (observed) | Excluded | | Measurement reliability | |
| Bulging fontanelles (observed) | Included | | | 2 |
| Mobility (observed) | Excluded | | Measurement reliability | |
| Focal neurology acute (observed) | Excluded | | Measurement reliability | |
| Confusion (observed) | Excluded | | Measurement reliability | |
| Lethargy (AVPU) (observed) | Included | | | 1 |
| Blantyre Coma Scale <5 (observed) | Excluded | | Measurement reliability | |
| **Infection** | | | | |
| Fever | Discussion | Excluded | Measurement reliability | |
| Suspected measles | Excluded | | Measurement reliability | |
| History of measles | Excluded | | Measurement reliability | |
| Ear pain | Discussion | Excluded | Low perceived predictive value | |
| Ear discharge (ex. pus) | Discussion | Excluded | Low perceived predictive value | |
| Tender swelling behind the ear | Discussion | Excluded | Low perceived predictive value | |
| Otitis media | Excluded | | Low perceived predictive value | |
| Purulent drainage eyes | Excluded | | Low perceived predictive value | |
| Conjunctivitis | Excluded | | Low perceived predictive value | |
| Rash | Included | | | 2 |
| Skin pustules | Discussion | Excluded | Low perceived predictive value | |
| Hardening of skin | Excluded | | Low perceived predictive value | |
| Jaundice | Discussion | Included | | 1 |
| Umbilicus - red | Included | | | 2 |

| | | | | |
|---|---|---|---|---|
| Umbilicus - draining (pus) | Included | | | 2 |
| Look for ulcers or white patches in the mouth (oral thrush) | Included | | | 2 |
| Risus sardonicus | Excluded | | Low perceived predictive value | |
| Tetanic spasms, spasticity | Excluded | | Low perceived predictive value | |
| Signs of local bacterial infections | Excluded | | Measurement reliability | |
| **GI/GU** | | | | |
| Diarrhea (reported) | Included | | | 1 |
| Blood in stool (dysentery) (reported) | Included | | | 2 |
| Vomiting (reported) | Included | | | 1 |
| Abdominal pain (reported) | Included | | | 2 |
| Abdominal distension (observed) | Included | | | 3* |
| Foul-smelling urine (reported) | Excluded | | Low perceived predictive value | |
| **Malnutrition** | | | | |
| Too small | Excluded | | Measurement reliability | |
| Swelling of both feet (peripheral edema) | Included | | | 2* |
| Visible severe wasting (marasmus) | Included | | | 3* |
| **Trauma/Surgical** | | | | |
| Malformation | Excluded | | Measurement reliability | |
| Trauma (or other surgical condition) | Included | | | 2 |
| Eye injury | Discussion | Excluded | Captured in "Trauma" variable | |
| Fracture | Discussion | Excluded | Captured in "Trauma" variable | |
| Dislocation | Discussion | Excluded | Captured in "Trauma" variable | |
| Hemorrhage | Discussion | Excluded | Captured in "Trauma" variable | |
| Severe Pain | Discussion | Included | | 3* |
| Burns | Included | | | 2 |

| | | | | |
|---|---|---|---|---|
| Poisoning | Included | | | 2 |
| **Other** | | | | |
| History of diabetes | Excluded | | Low perceived predictive value | |
| Sleep quality | Excluded | | Measurement reliability | |
| Smiling (older than 6 weeks) | Discussion | Excluded | Measurement reliability | |
| Parental concern | Discussion | Excluded | Measurement reliability | |
| **Laboratory Testing** | | | | |
| Hemoglobin (g/dL) | Included | | | 2 |
| Malaria blood smear | Included | | | 1 |
| Blood sugar measurement | Included | | | 2 |

*Consensus for tiering achieved post-discussion (Step 5).
