# Supplementary material for: Common data elements for predictors of pediatric sepsis: A framework to standardize data collection
Source: PLoS One. 2021 Jun 10;16(6):e0253051. doi: 10.1371/journal.pone.0253051 (PMC8192005; doi:10.1371/journal.pone.0253051)
Supplement: S2 Table — (PDF) [file pone.0253051.s004.pdf]

S4 Table. Additional predictor variables proposed in Step 3 (N=64). Displays decision to include (N=39) or exclude (N=25) from the common data element set, and exclusion reason or importance tier as applicable.

Variable	Status	Reason for exclusion	Tier
**Patient Characteristics**			
Admission within 6 months (reported)	Included		1
Number of times hospitalized within 6 months (reported)	Included		2*
Length of Illness prior to seeking help (days, reported)	Included		2*
Prior care for this illness	Excluded	Low perceived predictive value	
Mode of transport to hospital	Excluded	Low perceived predictive value	
History of fever	Included		1
**Pregnancy/Birth Details**			
Birth weight	Included		1
Gestational age	Included		1
Mode of delivery	Excluded	Low perceived predictive value	
Details of cord after birth	Excluded	Low perceived predictive value	
Number of antenatal care visits	Included		2*
**Sociodemographic Information**			
Primary caregiver	Included		1
Primary caregiver sex	Excluded	Low perceived predictive value	
Primary caregiver age	Excluded	Low perceived predictive value	
Primary caregiver marital status	Excluded	Low perceived predictive value	
Maternal age at first pregnancy	Excluded	Low perceived predictive value	
Mother acutely ill	Excluded	Low perceived predictive value	
Maternal chronic illness requiring ongoing treatment or follow- up (HIV, TB, mental illness, etc.)	Excluded	Low perceived predictive value	

Maternal substance use in last 30 days (check all that apply)	Excluded	Low perceived predictive value	
Maternal HIV treatment status	Excluded	Low perceived predictive value	
Non-exclusive breastfeeding details	Excluded	Low perceived predictive value	
Number of people in child's house	Excluded	Low perceived predictive value	
Cooking fuel	Excluded	Low perceived predictive value	
Cooking location	Excluded	Low perceived predictive value	
Lighting	Excluded	Low perceived predictive value	
Smoking	Excluded	Low perceived predictive value	
**Vitals**			
Modality of oxygen delivery	Included		2
Flow of oxygen delivered	Included		2
Fractional inspired oxygen	Included		2
**Clinical Signs/Symptoms**			
**Respiratory**			
Inability to feed because of respiratory problems (reported)	Included		2
Inability to sit or stand because of respiratory problems (reported)	Included		2
Deep breathing (observed)	Excluded	Measurement Reliability	
Cough (observed)	Included		1
Tracheal tug (observed)	Included		2
**Circulation/Perfusion**			
Lower limb temperature gradient	Included		2*
**Dehydration**			
Changes in urine colour	Excluded	Low perceived predictive value	
**Neurological**			
Irritability/restlessness (observed)	Included		2
Eye movement (Blantyre Coma Scale) (observed)	Excluded	Measurement Reliability	

Best motor response (Blantyre Coma Scale) (observed)	Excluded	Measurement Reliability	
Best verbal response (Blantyre Coma Scale) (observed)	Excluded	Measurement Reliability	
**Infection**			
Muscle tone	Excluded	Measurement Reliability	
TB contact	Excluded	Measurement Reliability	
**Laboratory Testing**			
HIV testing	Included		1
Lactate (mmol/L)	Included		2
Bilirubin measurement	Included		2
WBC	Included		2
pH	Included		3
Potassium (mmol/L)	Included		3
Base excess (mmol/L)	Included		3
Nasopharyngeal swab	Included		3
**Treatments Received Prior to Study**			
Receipt of antibiotics prior to recruitment	Included		2
Receipt of antipyretics prior to recruitment	Included		2
Receipt of IV fluids prior to recruitment	Included		2
Receipt of other medication prior to recruitment	Included		3
Route of antibiotics	Included		3
Type of antibiotic	Included		3
Time since last antibiotic	Included		3
Time since last antipyretic	Included		3
Type of antipyretic	Included		3
Route of medication	Included		3
Type of medication	Included		3

Visit to any healthcare provider prior to recruitment	Included		3
What type of healthcare provider	Included		3
Number of providers	Included		3

*Consensus for tiering achieved post-discussion (Step 5).
