# Supplementary material for: Common data elements for predictors of pediatric sepsis: A framework to standardize data collection
Source: PLoS One. 2021 Jun 10;16(6):e0253051. doi: 10.1371/journal.pone.0253051 (PMC8192005; doi:10.1371/journal.pone.0253051)
Supplement: Attachment — Submitted filename: Response to PLOS ONE revieweres 26APR21.docx [file pone.0253051.s005.docx]

**COVER LETTER: RESPONSE TO ACADEMIC EDITOR AND REVIEWERS**

Thank you for all your helpful comments and suggestions!

**ACADEMIC EDITOR**

**1. Please ensure that your manuscript meets PLOS ONE's style requirements, including those for file naming. The PLOS ONE style templates can be found at**

**https://journals.plos.org/plosone/s/file?id=wjVg/PLOSOne_formatting_sample_main_body.pdf and**

[**https://journals.plos.org/plosone/s/file?id=ba62/PLOSOne_formatting_sample_title_authors_affiliations.pdf**](https://journals.plos.org/plosone/s/file?id=ba62/PLOSOne_formatting_sample_title_authors_affiliations.pdf)

We have ensured that the manuscript meets PLOS ONE's style requirements, including those for file naming.

**2. Please review your reference list to ensure that it is complete and correct. If you have cited papers that have been retracted, please include the rationale for doing so in the manuscript text, or remove these references and replace them with relevant current references. Any changes to the reference list should be mentioned in the rebuttal letter that accompanies your revised manuscript. If you need to cite a retracted article, indicate the article’s retracted status in the References list and also include a citation and full reference for the retraction notice.**

The reference list is complete and correct.

**3. Please provide additional details regarding participant consent to collect personal data, including email addresses, names, or phone numbers. In the Methods section, please ensure that you have specified how consent was obtained and how the study met relevant personal data and privacy laws. If data were collected anonymously, please include this information.**

This study did not require participant consent or collection of personal data. All expert contributors are members of the Pediatric Sepsis CoLab and are listed as authors on the manuscript (see author contribution section). There was no anonymity since data collection involved active group discussions among the working group members.

We have added the following statement below the METHODS heading: *Ethics approval was obtained from the Child and Women’s Research Ethics Board at the University of British Columbia (H17-01893). All expert contributors are members of the Pediatric Sepsis CoLab and voluntary involvement in the Delphi process implied consent.*

**4. Thank you for stating the following in the Acknowledgments Section of your manuscript:**

**[AC is supported by a Wellcome Trust Doctoral Training Fellowship. SA was supported by the Initiative to Develop African Research Leaders (IDeAL) Wellcome Trust award (# 107769).]**

**We note that you have provided funding information that is not currently declared in your Funding Statement. However, funding information should not appear in the Acknowledgments section or other areas of your manuscript. We will only publish funding information present in the Funding Statement section of the online submission form.**

**Please remove any funding-related text from the manuscript and let us know how you would like to update your Funding Statement. Currently, your Funding Statement reads as follows:**

**[The authors received no specific funding for this work.] Please include your amended statements within your cover letter; we will change the online submission form on your behalf.**

We have removed all funding information from the acknowledgements section.

The acknowledgements statement has been updated to the following:

*We would like to thank contributing members of the Pediatric Sepsis CoLab for their time, effort, and enthusiasm towards development of the common data element guidelines.*

Please update the funding statement to the following:

*AC is supported by a Wellcome Trust Doctoral Training Fellowship. SA was supported by the Initiative to Develop African Research Leaders (IDeAL) Wellcome Trust award (# 107769). TK receives salary support from the National Institute of Allergy and Infectious Diseases, USA (K23AI1440.29).*

**5. We noted in your submission details that a portion of your manuscript may have been presented or published elsewhere. [Figure 1 (the systematic review process) was also published in our related manuscript. It is not dual publication because are just using the results of a literature review we conducted in the previous publication for a new purpose. This is further detailed in the cover letter. ] Please clarify whether this publication was peer-reviewed and formally published. If this work was previously peer-reviewed and published, in the cover letter please provide the reason that this work does not constitute dual publication and should be included in the current manuscript.**

Figure 1 is a file that outlines the search strategy for our previously conducted systematic review that was published in PLOS ONE. The results from our previous systematic review were used to compile the initial list of predictors to be used as input for the Delphi process in this current study. We included the search strategy in both our previously published manuscript, as well as this manuscript as we thought it was important for readers to understand the process behind the systematic review. This is not dual publication as the two publications have entirely different purposes, methodology, and results. The purpose of this current paper was to have a group of experts collaborate to create common data element guidelines in order to increase harmonization and data compatibility in the pediatric sepsis research community. No previous work has been published on this topic - this manuscript would be the first attempt at creating and presenting a standardized framework for data collection in the field of pediatric sepsis prediction.

**6. Please ensure that you refer to Figure 1 in your text as, if accepted, production will need this reference to link the reader to the figure.**

This has been done.

**7. Please include captions for ALL your Supporting Information files at the end of your manuscript, and update any in-text citations to match accordingly. Please see our Supporting Information guidelines for more information:** [**http://journals.plos.org/plosone/s/supporting-information**](about:blank)**.**

This has been done.

**REVIEWER #1**

**The article presents a collection of predictors of pediatric sepsis and describes the process how this set was selected. The selection is based on expert’s opinions, not analysing data. The aim is to make the selected data elements as a standardised way to collect pediatric sepsis data. This would increase compatibility across different studies on the topic. The topic of the study is definitely important, and results have a clear impact.

The research methodology is based on systematic literature review and domain experts’ knowledge through Delphi method. Finally, 116 data elements were accepted. The methodology is very well described and suitable for the study. However, the article does not describe whether any data collection has already done. Even a limited evaluation by using existing data would clearly improve the study.

Naturally, predictive power of the data items is difficult to measure since it would require a large amount of data and it also depends on the research questions at hand. If any data is already available, some data quality measures could have been computed. These measures could include basic descriptive statistics such as the number of observations per patient, missing values, correlations, and distributions. This statistical validation could have been an additional step in used methodology, but it would also be an additional validation step for the defined set of data items.**

Thank you for your feedback!

We agree that statistical evaluation of predictors would greatly strengthen the guidelines. We have used these data elements in the design of two independent, ongoing studies led by members of our working group.^1,2^ We will be publishing the predictive power of each of these elements once data has been collected. However, the purpose of this paper was to provide a wider group of researchers in the field of pediatric sepsis a proposed set of guidelines to be implemented into case report forms for future studies in order to standardize data collection and facilitate data sharing. Such data sharing would enable predictor validation as proposed but would not be possible without a set of standardization guidelines such as ours. We have been approached by numerous members of the Pediatric Sepsis CoLab inquiring about use of the guidelines for upcoming studies in the design phase. Data from studies such as these may be used validate various aspects of our predictor selection in multiple settings during the next iteration of our guidelines. Publication of our guidelines at this time will help disseminate knowledge of its existence, resulting in faster data accumulation for this validation process.

^1^Mawji A, Li E, Komugisha C, Akech S, Dunsmuir D, Wiens MO, Kissoon N, Kenya-Mugisha N, Tagoola A, Kimutai D, Bone JN, Dumont G, Ansermino JM. Smart triage: triage and management of sepsis in children using the point-of-care Pediatric Rapid Sepsis Trigger (PRST) tool. BMC Health Serv Res. 2020 Jun 3;20(1):493. doi: 10.1186/s12913-020-05344-w

^2^Chandna A, Aderie EM, Ahmad R, et al. Prediction of disease severity in young children presenting with acute febrile illness in resource-limited settings: a protocol for a prospective observational study. BMJ Open 2021;11:e045826. doi:10.1136/ bmjopen-2020-045826

**The manuscript mentions that the aim is to facilitate the design of efficient data collections tools. Could you explain better what kind of tools these could be? In Conclusion, risk prediction tools are also mentioned. Are these two different sets of tools?**

By efficient data collection tools, we mean the common data element guidelines can be used to create simple, standardized case report forms to be used for data collection in future studies. By risk prediction tools, we mean risk prediction algorithms that are derived and validated using compatible shared data, made possible by adoption of our guidelines. These are different types of “tools.” As stated in our abstract and introduction, then re-iterated in our discussion: Our guidelines aim to directly facilitate the former, but in doing so will indirectly catalyze the latter.

We understand the confusion as likely arising from the proximity of the terms “efficient data collection tools” and “risk prediction tools” in the abstract. We have modified these two terms throughout the paper to read “efficient data collection methods” and “risk prediction algorithms” to avoid confusion of the two. We have also clarified the meaning of “efficient data collection methods” with an example: a low-cost electronic case report form on a mobile application.

**Other comments:
-Two tables S2 and S3 have the same title “Common data elements for predictors of pediatric sepsis: A framework to standardize data collection”**

The supplementary files have been renamed for clarity:

**S3 Table:** Preliminary list of predictor variables compiled through literature review (N=144). Displays decision to include (N=77) or exclude (N=67) from the common data element set, and reason for exclusion where applicable. *Consensus for tiering achieved post-discussion (Step 5).

**S4 Table:** Additional predictor variables proposed in Step 3 (N=64). Displays decision to include (N=39) or exclude (N=25) from the common data element set, and exclusion reason or importance tier as applicable.

*Consensus for tiering achieved post-discussion (Step 5).

**-On these tables a reason for exclusion can be “low perceived predictive values”. Is also based only on expert’s opinions or statistical analysis?**

As described in the methodology, a Delphi processes using expert consensus was used to exclude predictor variables, no quantitative statistical analysis was conducted. This study was based on collaborative effort from experts in the field of pediatric sepsis to create widely accepted guidelines which will maximize uptake in future studies.

**REVIEWER #2**

**Abstract:**

**-I really like how the amount of time and material resources to reliably collect the predictor in resource-limited settings was considered -- this is extremely important.**

Thank you.

**-Conclusion line 72: replace "remains" with "remain"**

Thank you, this has been corrected.

**Introduction:**

**-Lines 102 and 103: Please standardize how you present "data-driven" vs "data driven", as you spell it with and without a hyphen.**

Thank you, this has been corrected. Presented as “data driven” throughout the manuscript.

**-Line 105: Specify which settings you are referring to here. Low-resource settings?**

Clarified. Referring to resource-limited settings.

**-Line 106: Improving healthcare delivery seems vague here compared to the following two examples of timely treatment and early referral. Perhaps clarify a bit or omit.**

This has been clarified: *“Early identification of the at-risk patient can improve healthcare delivery by facilitating timely administration of treatment before significant deterioration occurs, and early referral when a higher level of care is required.”*

**-Line 113: Consider adding a source here.**

Reference included: *Dowell SD, Blazes D, Desmond-Helman S. Four steps to precision public health. Nature 2016; 540: 189-191. doi 10.1038/540189a*

**-Lines 121-123: Use of commas is confusing here, and it ends up being a run-on sentence. I would use ";" to help organize this sentence.**

This has been clarified: “*By increasing consistency of data collected across studies common data elements can facilitate data aggregation, meta-analyses, cross-study comparisons, and simplify training operations to increase overall efficiency and quality of data collection (7).”*

**-Line 125: For those unfamiliar with the PS2, I would suggest adding "a subgroup within the Pediatric Sepsis CoLab" after workgroup here. I know you go into more detail in the methods, but I would at least add that here in the Intro.**

This has been included: *“The Pediatric Sepsis Predictor Standardization (PS2) working group, a subgroup within the Pediatric Sepsis CoLab, has generated a set of common data elements for predictors of pediatric sepsis to serve as a standardized framework for data collection in future studies.”*

**-Last paragraph: Clarify what the objective of this current paper is. Is it the first sentence? I would suggest making this more clear by stating "the objective of this study" or something like that.**

The last paragraph has been clarified:

*The purpose of this study was to create a universally accepted living framework to increase standardization of data collection in future studies of pediatric sepsis prediction. The Pediatric Sepsis Predictor Standardization (PS2) working group, a subgroup within the Pediatric Sepsis CoLab, has collaborated to develop a set of common data elements for predictors of pediatric sepsis at the point of triage in resource-limited settings. Standardized outcomes are being addressed by a related working group (9) (10). Routine use of the framework within the pediatric sepsis research community would promote an environment of collaboration, improve compatibility of datasets across studies, and yield robust, high quality sources of data that can be used to develop powerful risk prediction tools and support clinical decision making.*

**Methods:**

**-Objective section: This seems a bit repetitive with the objective paragraph in the Intro. Perhaps include this there, instead, and cut where redundant?**

This section has been removed and combined with the last paragraph in the introduction (see above).

**-Line 173: Please keep past/present tense consistent throughout as past tense. Here please say "may have yielded".**

Thank you, this has been corrected.

**-Line 174: Also, keep in either active or passive voice. This whole paragraph is in passive voice but the last sentence switches to active voice.**

Thank you, this has been corrected.

**-Lines 188-190: Consider here the use of ";" rather than "," as otherwise this sentence is hard to follow.**

Thank you, this has been corrected.

**-Lines 231-232: For those unfamiliar with the Delphi method, perhaps clarify what "80% threshold required for consensus". Does that mean that 80% of experts had to agree that it was important enough to be included?**

Predictors to be included in the common data element guidelines had already been selected at this point (in Step 2). This has been removed as it was d not relevant at this stage in the Delphi process.

**-IRB approval was not discussed and should be included.**

We have added a statement regarding IRB approval below the METHODS heading: *Ethics approval was obtained from the Child and Women’s Research Ethics Board at the University of British Columbia (H17-01893). All expert contributors are members of the Pediatric Sepsis CoLab and voluntary involvement in the Delphi process implied consent.*

**-If possible to add in a little bit about the experts, like what countries they represent, that would be very interesting.**

We have added these details in the PS2 working group section: *The PS2 working group is currently comprised of six experts in pediatric sepsis, with representation from Canada, the United States of America, East Africa, and Cambodia; and clinical expertise including pediatric critical care, infectious disease management, and anesthesia.*

**Results:
- Line 277: Missing a "(" in front of S2 Table.**

Thank you, this has been corrected.

**- Line 279-280: Most common reasons for exclusion "were".**

Thank you, this has been corrected.

**- Line 282: Please standardize spacing before and after "=" as you include spacing here but not elsewhere.**

Thank you, this has been corrected.

**- Lines 331-332: Although I understand the concept of branching logic being maximally harnessed by using electronic data collection, in many low-resource settings this is not a possibility. Is it also appropriate to do this on traditional paper methods?**

Branching logic could still help in settings where traditional paper methods are being used, as the logic could be displayed as prompts on case report forms. However, it would be less efficient as it would require the data collection team to manually follow the prompts and skip questions where appropriate. It is significant to note that very simple technology such as mobile devices can be used to implement a digital data collection tool. This technology is low-cost and used pretty much universally at this point in society.

**- Notes section: I might consider moving this to where you discuss Table 2 as otherwise the reader is jumping around quite a bit.**

Corrected. Reformatted the paper to have a section “Features of the guidelines” with all features described in the order they appear in the accompanying table (Table 1).

**- Standard operating protocols section: This seems a little out of place. I would either cut or elaborate beyond one simple sentence. Perhaps add an example to make this section more relevant and robust.**

We have expanded on this section: *Standard operating protocols* *have been developed to provide additional standardization in variables with more involved physical exam procedures, such as anthropometric measurements and vital signs. These are available as supplementary material alongside the common data element guidelines (20).*

**- Feedback survey: How are the suggestions on the survey incorporated into the common data element set? Are they incorporated immediately or at certain time points? Are the just added in or does consensus have to be achieved on discussion? Perhaps provide some details on this here.**

They will serve as input to future repetitions Delphi process, which is repeated every two years (see Discussion, Adoption and Use).

Details have been included in the feedback survey section: *A web-based survey is available through the Pediatric Sepsis CoLab for researchers to propose new candidate predictors or suggest modifications to existing variables (23). Feedback will be taken into account as input into future repetitions of the Delphi process to process to determine inclusion in updated iterations of the common data element set.*

**- I may have missed this, but where is the list of the 116 predictors? Perhaps be more clear about where to find these.**

Included right below the RESULTS heading: *The final set of 116 common data elements and supplementary materials are published and freely available on the Pediatric Sepsis CoLab Dataverse (20).*

**Discussion:
- Summary paragraph: This is well written, but it is overall restating a lot of what you have stated above already. I would try to reduce redundancy and streamline a bit if possible here.**

Deleted aspects to reduce redundancy.

**- Adoption and use: This paragraph is in future tense, but shouldn't it be past tense since you have already done this? Also, a lot of these 2 paragraphs should go above in the results rather than the discussion. A lot of it is redundant and elaborating on logistics of accessing the Dataverse, so I would say include above where you talk about Dataverse.**

Corrected. Thank you for bringing this to our attention.  **Conclusion:
- Final sentence lines 441-444: Instead of saying "This was with the intent..." consider something like "This will enable collaboration", which looks towards the future and proves the benefit of the study.**

Thank you. The conclusion has been updated: *This will enable collaboration and coordination in the pediatric sepsis research community and strengthen the quality of risk prediction models for pediatric sepsis to support clinical decision making at the point of triage.*

**REVIEWER #3**

**This is well-written and appropriately describes the Delphi process used. The expert consensus group is internationally representative but is small in number (6 experts). I have no major concerns.

Table 1 is confusing - it might be more helpful to include a sample with variables included, and to describe the significance of bolding and colors in the footnotes. Tables 2 and 3 are more clear.**

Thank you for your comments. We agree that Table 1 is confusing. We have revised Table 1 as per your suggestions by including sample variables and explaining the significance of bolding and colors in the foot notes.
